# Next Generation MUT-MAP, a High-Sensitivity High-Throughput Microfluidics Chip-Based Mutation Analysis Panel

**DOI:** 10.1371/journal.pone.0090761

**Published:** 2014-03-21

**Authors:** Erica B. Schleifman, Rachel Tam, Rajesh Patel, Alison Tsan, Teiko Sumiyoshi, Ling Fu, Rupal Desai, Nancy Schoenbrunner, Thomas W. Myers, Keith Bauer, Edward Smith, Rajiv Raja

**Affiliations:** 1 Oncology Biomarker Development, Genentech Inc., South San Francisco, California, United States of America; 2 Chemistry and Innovation Technology, Pleasanton, California, United States of America; 3 Program in Core Research, Roche Molecular Systems Inc., Pleasanton, California, United States of America; Deutsches Krebsforschungszentrum, Germany

## Abstract

Molecular profiling of tumor tissue to detect alterations, such as oncogenic mutations, plays a vital role in determining treatment options in oncology. Hence, there is an increasing need for a robust and high-throughput technology to detect oncogenic hotspot mutations. Although commercial assays are available to detect genetic alterations in single genes, only a limited amount of tissue is often available from patients, requiring multiplexing to allow for simultaneous detection of mutations in many genes using low DNA input. Even though next-generation sequencing (NGS) platforms provide powerful tools for this purpose, they face challenges such as high cost, large DNA input requirement, complex data analysis, and long turnaround times, limiting their use in clinical settings. We report the development of the next generation mutation multi-analyte panel (MUT-MAP), a high-throughput microfluidic, panel for detecting 120 somatic mutations across eleven genes of therapeutic interest (*AKT1*, *BRAF*, *EGFR*, *FGFR3*, *FLT3*, *HRAS*, *KIT*, *KRAS*, *MET*, *NRAS*, and *PIK3CA*) using allele-specific PCR (AS-PCR) and Taqman technology. This mutation panel requires as little as 2 ng of high quality DNA from fresh frozen or 100 ng of DNA from formalin-fixed paraffin-embedded (FFPE) tissues. Mutation calls, including an automated data analysis process, have been implemented to run 88 samples per day. Validation of this platform using plasmids showed robust signal and low cross-reactivity in all of the newly added assays and mutation calls in cell line samples were found to be consistent with the Catalogue of Somatic Mutations in Cancer (COSMIC) database allowing for direct comparison of our platform to Sanger sequencing. High correlation with NGS when compared to the SuraSeq500 panel run on the Ion Torrent platform in a FFPE dilution experiment showed assay sensitivity down to 0.45%. This multiplexed mutation panel is a valuable tool for high-throughput biomarker discovery in personalized medicine and cancer drug development.

## Introduction

Biological markers, or biomarkers, have been defined as “any substance, structure or process that can be measured in bio-specimen and which may be associated with health-related outcomes” [1]. Currently biomarkers are being used for prognostic, diagnostic, and predictive purposes in the field of oncology and as such play a vital role in personalized medicine. Biomarkers can be used to determine subsets of a population that may or may not respond to drug treatment/therapy and can even be used to prescreen patients in clinical trials. The reliable detection and validation of these markers is therefore essential.

In the last ten years, developments in genome-wide analytic methods have made the profiling of gene expression and genetic alternations of the cancer genome possible. By determining the molecular profile of a tumor (both mutational status and gene expression), a patient's disease can be characterized. This information can then be used to determine which course of treatment a patient should follow. A recent example of such targeted therapy is the development of ZELBORAF for treatment of patients whose unresectable or metastatic melanoma harbors a *BRAF* V600E mutation [2]. A companion diagnostic assay was developed with this drug to screen patients, allowing only those patients whose tumors were biomarker positive to receive the treatment. Somatic mutations, therefore, can serve as tumor specific biomarkers, allowing for the use of targeted therapies.

One of the biggest challenges in using clinical samples for biomarker detection is the fact that most tumor biopsies are formalin-fixed and paraffin-embedded (FFPE) for long term storage of the tissue [3]. This treatment leads to lower yield and quality of isolated genomic DNA (gDNA) from the samples due to cross-linking and fragmentation.

Characterization of the cancer genome by next generation sequencing (NGS) methods have emerged, ignited by the increased understanding of somatic alternations in cancer and their value in the development of personalized therapeutics. However, NGS lacks the analytical sensitivity and quantitative performance required for mutation detection in FFPE tissues. Furthermore, currently NGS requires larger DNA quantities for analysis, has complex and time consuming data analysis pipelines, and involves high costs, all of which makes NGS impractical for routine clinical use.

We previously developed a mutation multi-analyte panel (MUT-MAP) that allowed for the detection of 71 mutations across six oncogenes. This panel utilized the Fluidigm microfluidics technology which allowed for simultaneous detection of these mutations in a single sample. We report here the development and validation of the next generation MUT-MAP, a high-throughput platform that can now detect 120 hotspot mutations in eleven genes (*AKT1*, *BRAF*, *EGFR*, *FGFR3*, *FLT3*, *HRAS*, *KIT*, *KRAS*, *MET*, *NRAS*, and *PIK3CA*) based on allele specific PCR (AS-PCR) and Taqman technologies. Analysis of 88 samples can be completed in one day with as little as 2 ng of high quality gDNA or 100 ng of gDNA derived from FFPE tissues. By multiplexing our assays, less precious sample is required, resulting in a robust and easy to interpret data output.

The mutations detected in this panel are found in various types of cancers and the genes encode proteins of therapeutic interest. For example, bladder cancer has a 44% frequency of mutations in *FGFR3*,13% in *RAS* oncogenes (*HRAS*, *KRAS*, or *NRAS*), and 13–27% in *PIK3CA*. These mutations are currently being validated as potential diagnostic biomarkers for patient stratification in clinical trials [4]. Mutations in *FLT3* lead to constitutively active FLT3 which can then act in a ligand-independent manner in leukemia [5]. *KIT* mutations have been implicated in several cancers including melanoma [6] and gastrointestinal stromal tumors [7]. *MET* mutations are prevalent in hereditary and sporadic papillary renal cell carcinoma [8], head and neck carcinoma [9], and non-small cell and small cell lung cancer [10].

The updated MUT-MAP microfluidics system continues to provide a cost-effective, high-sensitivity, and high-throughput platform for exploratory analysis of predictive and prognostic biomarkers in clinical trial samples. It offers a means of detecting a wide range of mutations in a panel of eleven therapeutically relevant genes. The MUT-MAP system reported here can be used to analyze somatic mutations with very small amounts of gDNA from poor quality, archived FFPE tissues and could be used for exploratory biomarker analysis supporting the development of tools for predictive and prognostic assessment of various cancers.

## Materials and Methods

### Microfluidics

The updated MUT-MAP panel was run on the BioMark platform (Fluidigm Corp.) using a 96.96 dynamic array as described previously [11] with a few alterations. Preamplified DNA combined with qPCR reagents and 10× assays mixed with the Fluidigm 20× sample loading reagent (Fluidigm Corp.) were loaded onto the chip as per the manufacturer's protocol. All newly added assays were allele-specific PCR (AS-PCR) assays which utilized an engineered *Thermus specie* Z05 DNA polymerase (AS1) and primers to allow for allelic discrimination between the wild-type and mutant sequence. [12], [13] An exon specific probe was used in all assays.

### DNA Preamplification

DNA preamplification procedures were performed as described previously [11]. Briefly, DNA was preamplified in a 10 µl reaction for 20 cycles in the presence of a preamplification primer cocktail mix (Table S1 shows sequences of newly added primers) and 1× ABI Preamp Master Mix (Applied Biosystems; Foster City, CA). All samples were exonuclease treated after PCR amplification to remove the remaining primers before being loaded onto the chip. Exonuclease I (16 U) (New England Biolabs; Ipswitch, MA) in exonuclease reaction buffer and nuclease-free water were added to each 10 µl PCR amplification and incubated at 37°C for 30 min followed by a 15 min incubation at 80°C for enzyme inactivation. Samples were then diluted four-fold in nuclease-free water and stored at 4°C or −20°C until needed.

A positive control was prepared in bulk by amplification of a cocktail of relevant mutant plasmids for all eleven genes in the presence of a wild-type human genomic DNA background; this positive control was run in triplicate on every chip for quality control purposes.

### Preparation of Reagents

All assays from the previous MUT-MAP were prepared as described previously [11]. Final primer and probe concentrations of 200 and 100 nM were used respectively for the newly designed custom AS-PCR assays which were added to the panel. These assays are currently under development at Roche Molecular Systems, Inc. (Pleasanton, CA).

A commercially available COBAS PIK3CA Mutation Test (Roche Molecular Systems) was modified to achieve compatibility with the two-color BioMark readout (FAM and VIC) for mutation detections in the *PIK3CA* gene.

All assays were prepared by diluting assays with the 20× sample loading buffer (Fluidigm Corp.). Diluted samples were mixed with AS1 qPCR master mix and run in duplicate by loading 5 µL into each well of a primed 96.96 Fluidigm Chip. The 96.96 dynamic array was loaded and then analyzed with the BioMark reader as previously described [11].

Data was analyzed and cycle threshold (C_T_) values were determined using the BioMark real-time PCR analysis software (Fluidigm Corp.) and automated mutation calls were determined using an algorithm based on the difference in C_T_ (ΔC_T_) values between wild-type and mutant assays for all AS-PCR assays.

### Eleven-Gene Mutation Panel

This MUT-MAP panel can screen 120 hotspot mutations across the *AKT1*, *BRAF*, *EGFR*, *FGFR3*, *FLT3*, *HRAS*, *KIT*, *KRAS*, *MET*, *NRAS*, and *PIK3CA* genes. The mutation coverage of additional content on this panel is presented in Table 1.

**Table 1:** Mutation Coverage Breakdown by Gene.

Eleven-Gene Mutation Coverage by AS-PCR Assays					
Gene	Mutation Count	Exon	Mutation ID	cDNA Mutation Position	Amino Acid Mutation Position
*PIK3CA*	17	1	746	263 G>A	R88Q
		4	754	1034 A>T	N345K
		7	757	1258 T>C	C420R
		9	760	1624 G>A	E542K
			12458	1634 A>C	E545A
			764	1634 A>G	E545G
			765	1635 G>T	E545D
			763	1633 G>A	E545K
			147	1636 C>G	Q546E
			766	1636 C>A	Q546K
			12459	1637 A>G	Q546R
			25041	1637 A>T	Q546L
		20	773	3129 G>T	M1043I
			776	3140 A>T	H1047L
			775	3140 A>G	H1047R
			774	3139 C>T	H1047Y
			12597	3145 G>C	G1049R
*HRAS*	11	2	480	34 G>A	G12S
			481	34 G>T	G12C
			483	35 G>T	G12V
			484	35 G>A	G12D
			487	37 G>A	G13S
			486	37 G>C	G13R
		3	496	181 C>A	Q61K
			499	182 A>G	Q61R
			498	182 A>T	Q61L
			503	183 G>C	Q61Hc
			502	183 G>T	Q61Ht
*FGFR3*	9	6	714	742 C>T	R248C
			715	746 C>G	S249C
		8	718	1118 A>G	Y373C
			716	1108 G>T	G370C
			17461	1111 A>T	S371C
			24842	1138 G>A	G380R
		13	719	1948 A>G	K650E
			720	1949 A>T	K650M
		15	24802	2089 G>T	G697C
*FLT3*	4	20	785	2503 G>C	D835H
			783	2503 G>T	D835Y
			784	2504 A>T	D835V
			787	2505 T>A	D835E
*MET*	4	2	710	1124 A>G	N375S
		14	707	3029 C>T	T1010I
		19	699	3743 A>G	Y1248C
			700	3757 T>G	Y1253D
*KIT*	8	11	1219	1669 T>C	W557R
			1221	1669 T>G	W557G
			1290	1727 T>C	L576P
		13	1304	1924 A>G	K642E
			12706	1961 T>C	V654A
		17	1311	2446 G>C	D816H
			1310	2446 G>T	D816Y
			1314	2447 A>T	D816V

### Assay Specificity and Sensitivity

Individual plasmids, each containing a single mutation correlating to each newly added assay on the 11-gene panel were used as samples to determine assay specificity and determine potential cross-reactivity between different hotspots.

Five linearized mutant plasmids were mixed to a final concentration of 4 ng/µL. The resulting mixes were diluted in either nuclease-free water or wild-type genomic DNA (Taqman Control Human Genomic DNA, Life Technologies, Cat# 4312660) where the genomic DNA concentration was kept constant at 10 ng. All of the samples were analyzed by the 11-gene panel along with a standard curve of wild-type human gDNA alone. Percentage of each mutation detected was calculated and the lower limit of detection (LLOD) of the assays in a genomic DNA background was determined for each assay evaluated. The samples diluted in nuclease-free water allowed for the assessment of assay linearity.

### Platform Validation

Mutation calls were validated using cell lines as well as FFPE tissues. Cell lines with known mutations reported in the literature were used to confirm the sensitivity and specificity of the assays. Further, a total of nine FFPE samples with known mutation status were mixed together with varying DNA inputs into seven Latin square mixes. The final DNA concentration of each mix was 40 ng/µl. These seven mixes were analyzed on MUT-MAP as well as by the SuraSeq500 panel on the Ion Torrent platform [14] in order to compare mutation calls and sensitivity levels of both platforms. The resulting data has been uploaded to the European Nucleotide Archive, http://www.ebi.ac.uk/ena/data/view/PRJEB5209.

## Results

### Panel Contents

To increase the coverage of our MUT-MAP platform, AS-PCR assays for *HRAS*, *FGFR3*, *FLT3*, *KIT*, *MET*, and *PIK3CA* were added (Table 1). The updated panel can now detect 120 somatic mutations across eleven genes of therapeutic interest for a single sample. By multiplexing assays and using two detection channels (FAM and VIC), we were able to consolidate all the assays onto a single Fluidigm microfluidics chip allowing for the simultaneous detection of 120 mutations in 44 samples.

### Mutant Control Formulation

**Figure 1:**
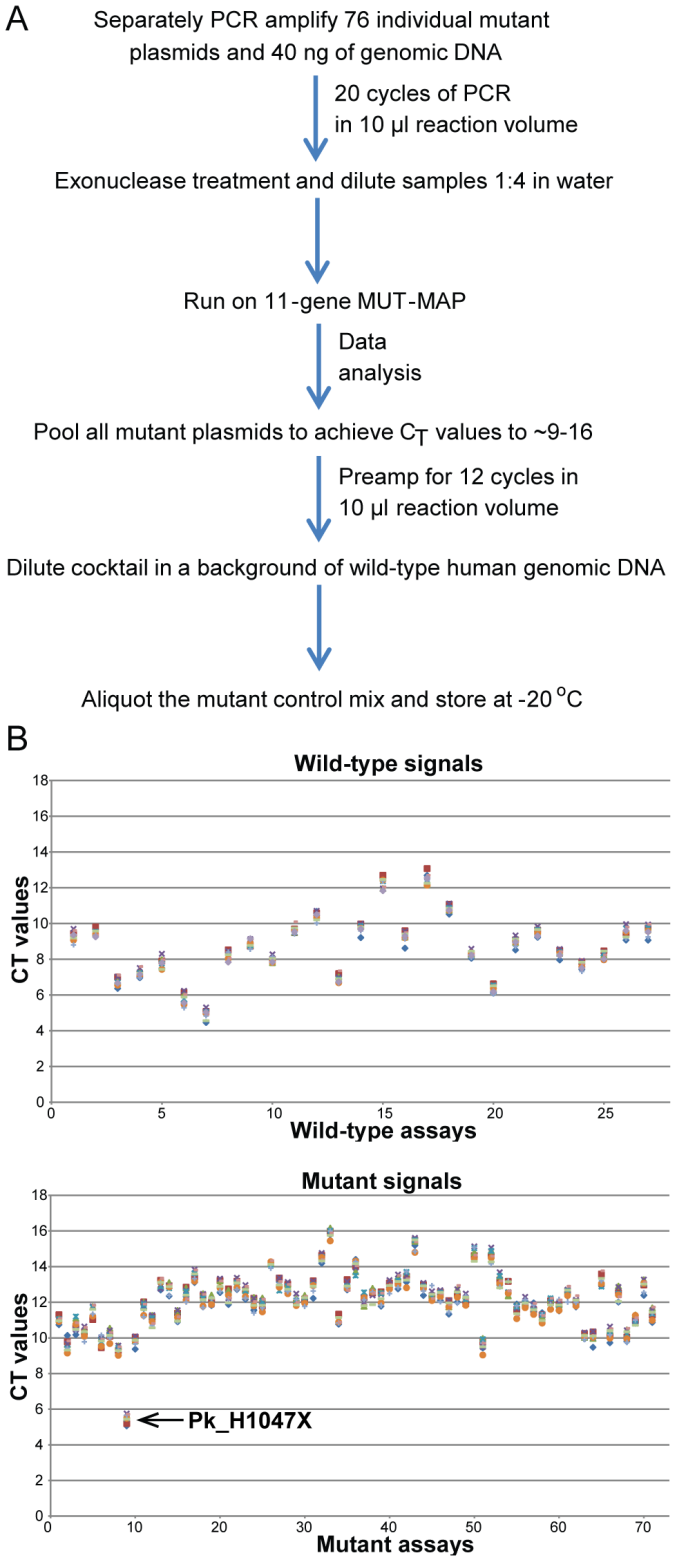
(**A**) Schematic diagram for the process of generating the positive control for MUT-MAP. (**B**) The positive control is a mixture of mutant plasmids and wild-type human genomic DNA. The positive control was created such that the resulting C_T_s range from 9–16 across all wild-type and mutant assays. Pk_H1047X covers multiple hotspot mutations resulting in a lower overall C_T_ as it is detecting more than one plasmid in the positive control.

A single control sample was formulated to be used as a positive control for every assay on MUT-MAP using the process described in Figure 1A. The positive control was generated by mixing mutant plasmids in the presence of a wild-type human genomic DNA background. The positive control was further preamplified and diluted to a concentration that resulted in C_T_ ranges from 9–16 across all wild-type and mutant assays (Figure 1B). This mutant control is included in every chip for quality control purposes.

### Assay Validation

A series of experiments were performed to validate the new assays added to the panel to ensure specificity and reproducibility. As described previously [11], a complete cross-reactivity analysis was conducted by screening a set of plasmids containing the mutant sequences against every assay on the panel. The C_T_ values generated from these experiments are shown in Tables 2 and 3 and Table S2. A C_T_ value of 30.0 indicates no amplification and that the specific mutation was not detected in that sample. Any C_T_ value lower than 30.0 indicate amplification and those values generated by mutation-specific assays on their corresponding mutant plasmid are indicated in bold (Tables 2 and 3).

**Table 2:** Cross-reactivity matrix for the newly added assays in *HRAS* and *PIK3CA*.

Assays	Plasmid controls											Controls	
	Hr_G12S	Hr_G12C	Hr_G12V	Hr_G12D	Hr_G13S	Hr_G13R	Hr_Q61K	Hr_Q61R	Hr_Q61L	Hr_Q61Hc	Hr_Q61Ht	gDNA	NTC
**Hr_ex2_WT**	**9.3**	**10.0**	**9.9**	**10.3**	**10.5**	**11.0**	30.0	30.0	30.0	25.0	30.0	**13.2**	30.0
**Hr_G12S**	**10.6**	21.9	22.5	24.8	21.4	21.3	30.0	30.0	30.0	30.0	30.0	25.0	30.0
**Hr_G12C**	20.8	**10.7**	23.4	24.3	22.4	23.1	30.0	30.0	30.0	30.0	30.0	25.2	30.0
**Hr_G12V**	30.0	30.0	**11.0**	22.9	22.8	22.4	30.0	30.0	30.0	30.0	30.0	26.6	30.0
**Hr_G12D**	30.0	22.2	20.7	**10.4**	23.4	22.9	30.0	30.0	30.0	30.0	26.5	30.0	30.0
**Hr_G13S**	20.4	20.8	30.0	30.0	**11.1**	25.0	30.0	30.0	30.0	30.0	30.0	24.2	30.0
**Hr_G13R**	30.0	30.0	30.0	30.0	30.0	**8.4**	30.0	30.0	30.0	30.0	30.0	30.0	30.0
**Hr_ex3_WT**	30.0	30.0	23.3	22.5	30.0	30.0	**8.4**	**8.6**	**8.7**	**7.9**	**8.8**	**10.6**	30.0
**Hr_Q61K**	30.0	30.0	30.0	30.0	30.0	30.0	**8.9**	24.4	30.0	19.6	22.3	21.3	30.0
**Hr_Q61R**	30.0	30.0	30.0	30.0	30.0	30.0	30.0	**9.2**	23.3	19.4	22.3	20.1	30.0
**Hr_Q61L**	30.0	30.0	30.0	30.0	30.0	30.0	30.0	23.1	**9.8**	21.6	24.2	22.3	30.0
**Hr_Q61Hc**	30.0	30.0	25.0	30.0	30.0	30.0	30.0	30.0	30.0	**8.8**	19.1	24.8	30.0
**Hr_Q61Ht**	30.0	30.0	30.0	30.0	30.0	30.0	30.0	30.0	30.0	18.8	**8.9**	22.4	30.0

**Table 3:** Cross-reactivity matrix for the newly added assays in *HRAS* and *PIK3CA*.

Assays	Plasmid controls									Controls	
	Pk_R88Q	Pk_N345K	Pk_C420R	Pk_E542K	Pk_E545K	Pk_Q546X	Pk_H1047R	Pk_M1043I	Pk_G1049R	gDNA	NTC
**Pk_ex1_WT**	**8.5**	8.5	8.9	8.2	7.9	30.0	30.0	30.0	25.7	**8.8**	30.0
**Pk_R88Q**	**15.7**	20.3	21.3	20.3	20.1	30.0	30.0	30.0	30.0	22.0	30.0
**Pk_ex4_WT**	9.3	**9.1**	9.7	8.8	8.5	25.1	30.0	30.0	30.0	**9.1**	30.0
**Pk_N345K**	21.3	**13.8**	20.8	20.5	20.2	30.0	30.0	30.0	30.0	25.4	30.0
**Pk_ex7_WT**	9.5	9.4	**9.6**	8.8	8.5	30.0	30.0	30.0	30.0	**8.2**	30.0
**Pk_C420R**	19.8	19.6	**14.7**	19.1	19.0	30.0	30.0	30.0	30.0	25.9	30.0
**Pk_ex9_WT**	9.0	8.9	9.4	**8.2**	**8.1**	**16.2**	30.0	30.0	30.0	**10.3**	30.0
**Pk_E542K**	22.1	22.3	22.7	**14.3**	21.6	30.0	30.0	30.0	30.0	23.6	30.0
**Pk_E545X**	19.8	20.0	20.5	19.5	**14.8**	30.0	30.0	30.0	30.0	23.9	30.0
**Pk_Q546X**	20.7	21.0	21.4	20.5	19.4	**20.2**	30.0	30.0	30.0	24.9	30.0
**Pk_ex9_WT**	9.0	8.9	9.4	8.2	8.1	30.0	**7.7**	**7.3**	**9.2**	**10.3**	30.0
**Pk_H1047X**	17.4	17.4	18.5	16.3	16.8	30.0	**11.0**	21.3	18.9	17.8	30.0
**Pk_M1043I**	20.4	20.8	21.7	19.7	19.6	30.0	22.2	**9.7**	24.8	21.7	30.0
**Pk_G1049R**	24.0	23.3	24.0	21.8	22.3	30.0	25.9	25.6	**10.1**	23.4	30.0

By utilizing the new AS-PCR assays, we were able to prevent the cross-reactivity found in certain instances on our previous panel (Tables 2 and 3). This highlights the specificity of our assays as some of the mutations are in the exact same position but have a single altered base, as in the case of Hr_G12S (position 34 G>A) and Hr_G12C (position 34 G>T) in Table 2.

### Platform Reproducibility

**Figure 2:**
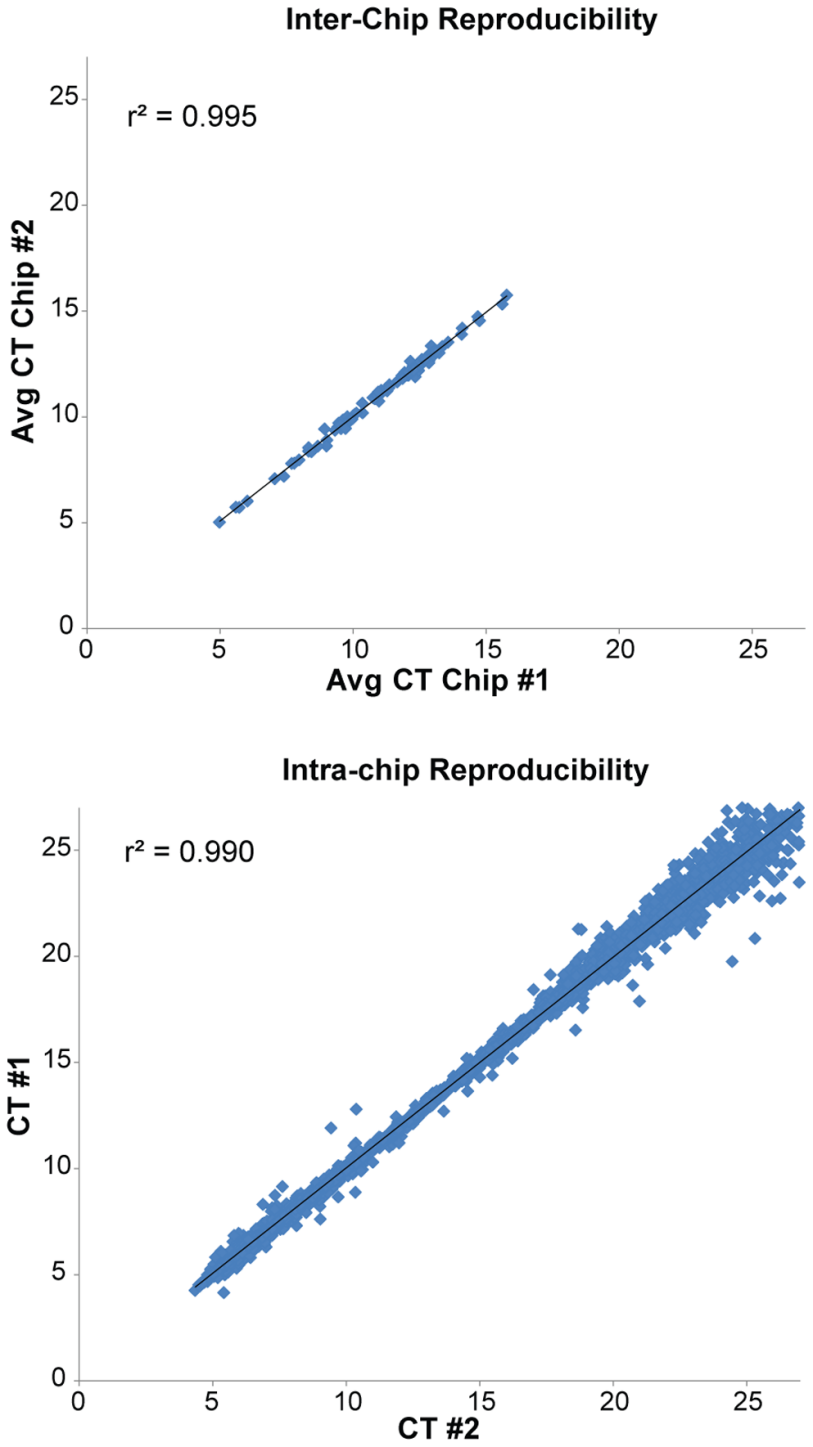
Quality control process for panel validation: Intra- and inter-chip reproducibility. MUT-MAP panel qPCR assays were run in duplicate and C_T_ outputs were plotted to determine both intra- and inter-chip reproducibility. Data for a typical mutation panel run are shown, with r^2^ values of 0.995 and 0.990 for inter- and intra-chip reproducibility, respectively.

The reproducibility of the mutation detection assays were evaluated by the comparison of duplicate experiments. The inter- and intra-chip variability in assay C_T_ values was examined as shown in Figure 2. Inter-chip reproducibility was accessed by directly comparing the C_T_ values of the mutant control between two chips and the Pearson correlation coefficient (r^2^) was calculated to be 0.995. A total of 5290 duplicate pairs were mapped on a scatter plot to determine the intra-chip reproducibility and the r^2^ value was found to be 0.990.

To insure that no variability was introduced by different operator analysis, data from a single MUT-MAP experiment was analyzed by three independent operators. The C_T_s for the mutant control were found to have an r^2^ value of 0.993 after multiple regression analysis (data not shown).

### Assay Sensitivity and Linearity

**Figure 3:**
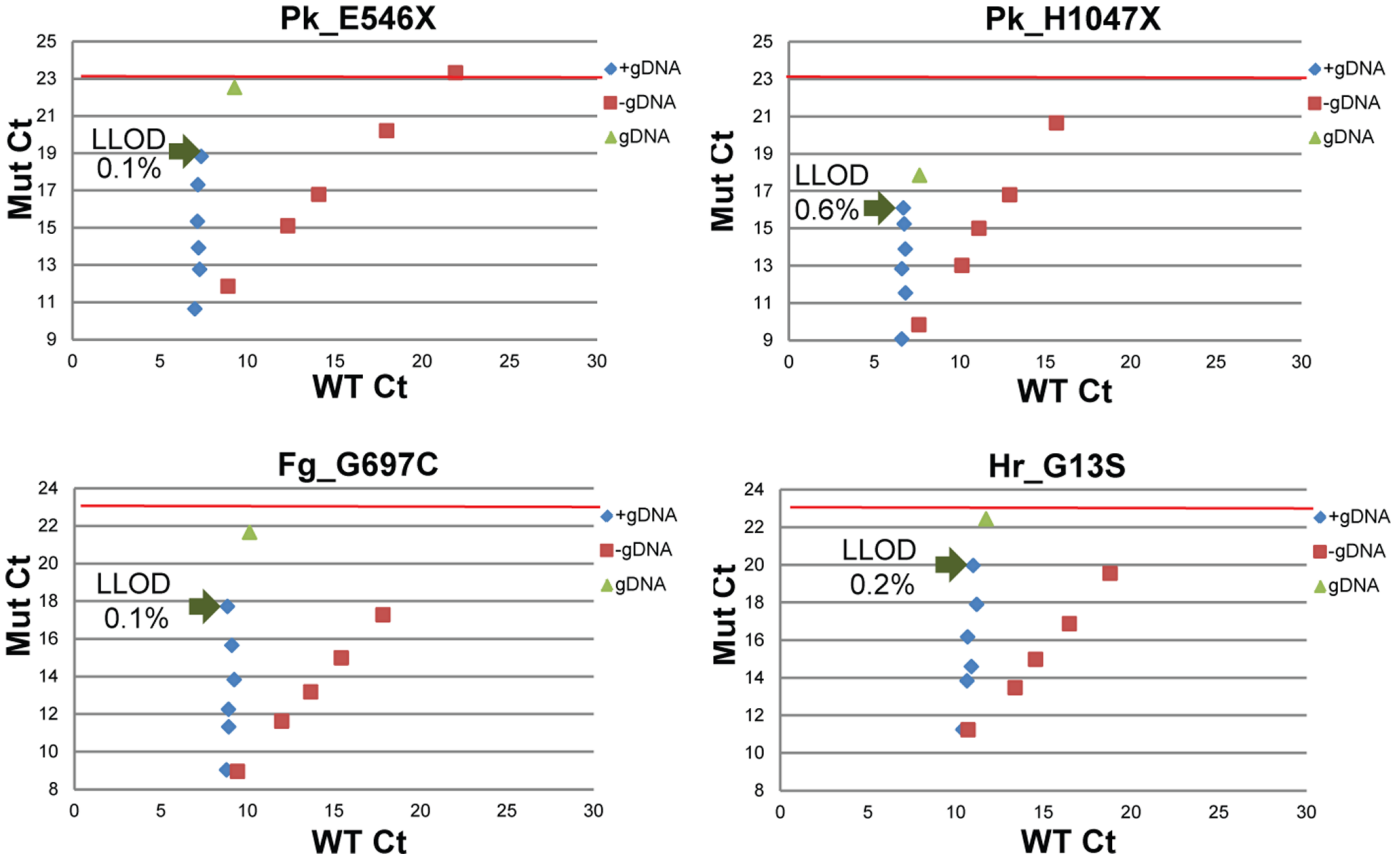
Evaluation of assay sensitivity. Linearized plasmids containing the mutant sequence were mixed and diluted into a background of wild-type genomic DNA from 50-0.1% mutant (blue diamonds). A sample containing 5% of the corresponding mutant plasmid with a wild-type genomic DNA background was diluted in nuclease-free water (red squares). Samples were run on the panel and assay sensitivity was determined. The C_T_ of wild-type genomic DNA alone is indicated by the green triangles.

When sensitivity of assays were assessed by diluting plasmids serially either in nuclease-free water or a constant wild-type genomic DNA background (10 ng), most assays showed a lower limit of detection (LLOD) of 0.1–0.2% with a few exceptions. A few examples of such sensitivity analysis are shown in Figure 3 and the remaining data is shown in Figure S1. The wild-type and mutant C_T_s for these samples are graphed in blue, clearly showing that in the constant wild-type genomic DNA background the indicated mutation can be detected down to LLOD of 0.1–0.2% with a few exceptions as marked in Figure 3. The plasmid diluted in nuclease-free water (red squares) illustrates excellent linearity of the assays.

### Validation of Cell Line Samples

For cell line samples, gene-specific custom algorithms were written, taking into account the control C_T_ and the mutant C_T_ values. Samples showing ΔC_T_<6 were determined as positive for the specific mutation.

Over 600 cell lines have been analyzed by the MUT-MAP to detect mutations across the eleven genes. Table 4 highlights some of the cell lines that were found to have mutations that were detected by the newly added assays. These mutation calls were compared with the published characteristics of these cell lines annotated in the COSMIC database [15].

**Table 4:** Correlation Between Mutation Calls in Cell Lines and Those Reported in the Literature.

		Eleven-Gene Mutation Panel										
Cosmic ID	Samples	*AKT1*	*BRAF*	*PIK3CA*	*NRAS*	*KRAS*	*EGFR*	*FGFR3*	*FLT3*	*HRAS*	*KIT*	*MET*
687505	C-33 A	MND	MND	**R88Q**	MND	MND	MND	MND	MND	MND	MND	MND
909757	SW 948	MND	MND	**E542K**	MND	**Q61L**	MND	MND	MND	MND	MND	MND
906824	Ca Ski	MND	MND	**E545K**	MND	MND	MND	MND	MND	MND	MND	MND
908138	MKN-1	MND	MND	**E545X**	MND	MND	MND	MND	MND	MND	MND	MND
924239	L-363	MND	MND	**E545X**	MND	MND	MND	MND	MND	MND	MND	MND
910698	BFTC-909	MND	MND	**E545K**	MND	MND	MND	MND	MND	MND	MND	MND
924100	22Rv1	MND	MND	**Q546X**	MND	MND	MND	MND	MND	MND	MND	MND
1752763	Detroit 562	MND	MND	**H1047X**	MND	MND	MND	MND	MND	MND	MND	MND
909698	RKO	MND	**V600E**	**H1047R**	MND	MND	MND	MND	MND	MND	MND	MND
1707559	MCAS	MND	MND	**H1047R**	MND	**G12D**	MND	MND	MND	MND	MND	MND
1707559	HEC-1-A	MND	MND	**G1049R**	MND	**G12D**	MND	MND	MND	MND	MND	MND
1576458	HEC-1-B	MND	MND	**G1049R**	MND	**G12D**	MND	MND	MND	MND	MND	MND
1740213	KMS-11	MND	MND	MND	MND	MND	MND	**Y373C**	MND	MND	MND	MND
909249	OPM-2	MND	MND	MND	MND	MND	MND	**K650E**	MND	MND	MND	MND
1339921	KYSE-30	MND	MND	MND	MND	MND	MND	MND	MND	**Q61L**	MND	MND
1752766	SCC-25	MND	MND	MND	MND	MND	MND	MND	MND	MND	MND	**N375S**
688093	Caov-4	MND	MND	MND	MND	MND	MND	MND	MND	MND	MND	MND
1436036	OVCAR-8	MND	MND	MND	MND	MND	MND	MND	MND	MND	MND	MND
909777	U-698-M	MND	MND	MND	MND	MND	MND	MND	MND	MND	MND	MND
1086323	BJAB	MND	MND	MND	MND	MND	MND	MND	MND	MND	MND	MND
1295511	SU-DHL-8	MND	MND	MND	MND	MND	MND	MND	MND	MND	MND	MND

MND, mutation not detected.

### Benchmarking Sensitivity of MUT-MAP with NGS

**Figure 4:**
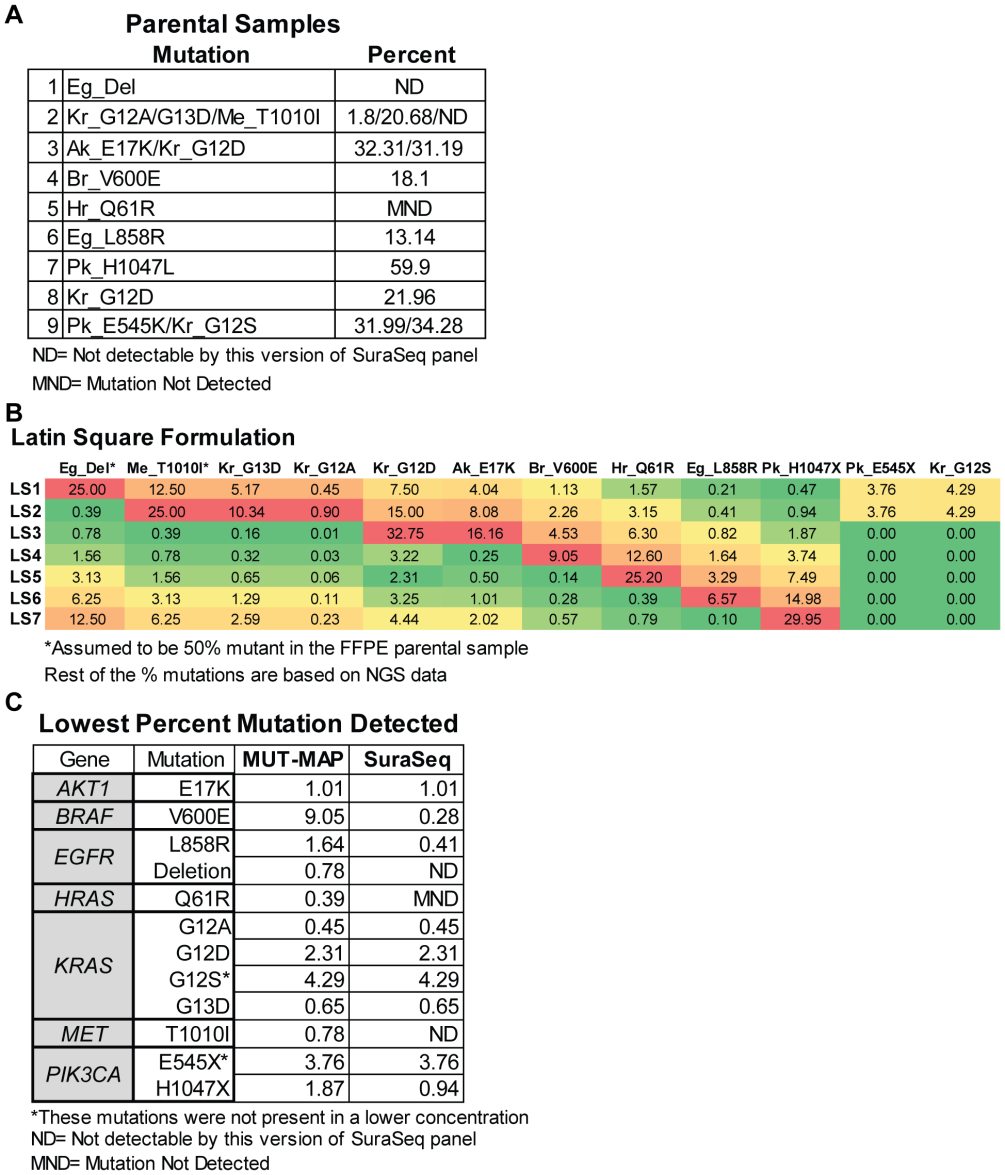
Comparison of the sensitivity of MUT-MAP and a next generation sequencing platform. (**A and B**) Nine FFPE samples with known mutation status were mixed together in varying concentrations following a Latin Square design to generate a seven-member Latin Square panel. The percentage of the mutant allele in each mix was calculated based on the mutant fraction of the parental samples as determined by analysis with the SuraSeq500 panel. For those mutations not detected by the NGS panel, 50% mutation in the parental sample was assumed. (**C**) The seven Latin Square samples were analyzed on MUT-MAP as well as by the SuraSeq500 panel on Ion Torrent in order to compare mutation calls and sensitivity levels of both platforms.

To assess the accuracy and sensitivity of the MUT-MAP, we compared it with a commonly used NGS platform. Seven Latin Square mixes were formulated by mixing nine different FFPE samples containing twelve hotspot mutations (*AKT1* E17K, *BRAF* V600E, *EGFR* deletion and L858R, *HRAS* Q61R, *KRAS* G12A, D, S and G13D, *MET* T1010I, and *PIK3CA* E545K and H1047L). When possible, the percentage of each mutation in the parental samples was determined by the SuraSeq500 panel (Figure 4A). Based on these percentages, the amount of each mutation in the seven Latin Square mixes were calculated and ranged from 0.14–32% (Figure 4B). By analyzing these samples on both platforms we were able to directly compare the sensitivity of twelve of our assays with the SuraSeq500 panel (Figure 4C).

MUT-MAP was able to detect down to a 1.87% mutation for *PIK3CA* H1047X while NGS detected down to 0.94%. For *BRAF* V600E, MUT-MAP utilizes a TaqMan assay which was found to be less sensitive than the SuraSeq500 panel (9.05% and 0.28% respectively). Both platforms showed similar sensitivity to the *AKT1* E17K mutation, as well as, the *KRAS* G12A and D, and G13D mutations. For the *PIK3CA* E545X and *KRAS* G12S mutations, both platforms were able to detect the lowest concentration present in our Latin Square mixes. The MUT-MAP panel also was able to detect *HRAS* Q61R down to a frequency of 0.39% while the SuraSeq500 panel did not detect the mutation at all in the Latin Square mixes or in the parental sample.

### Disease-Specific Prevalence Study Analyses

**Figure 5:**
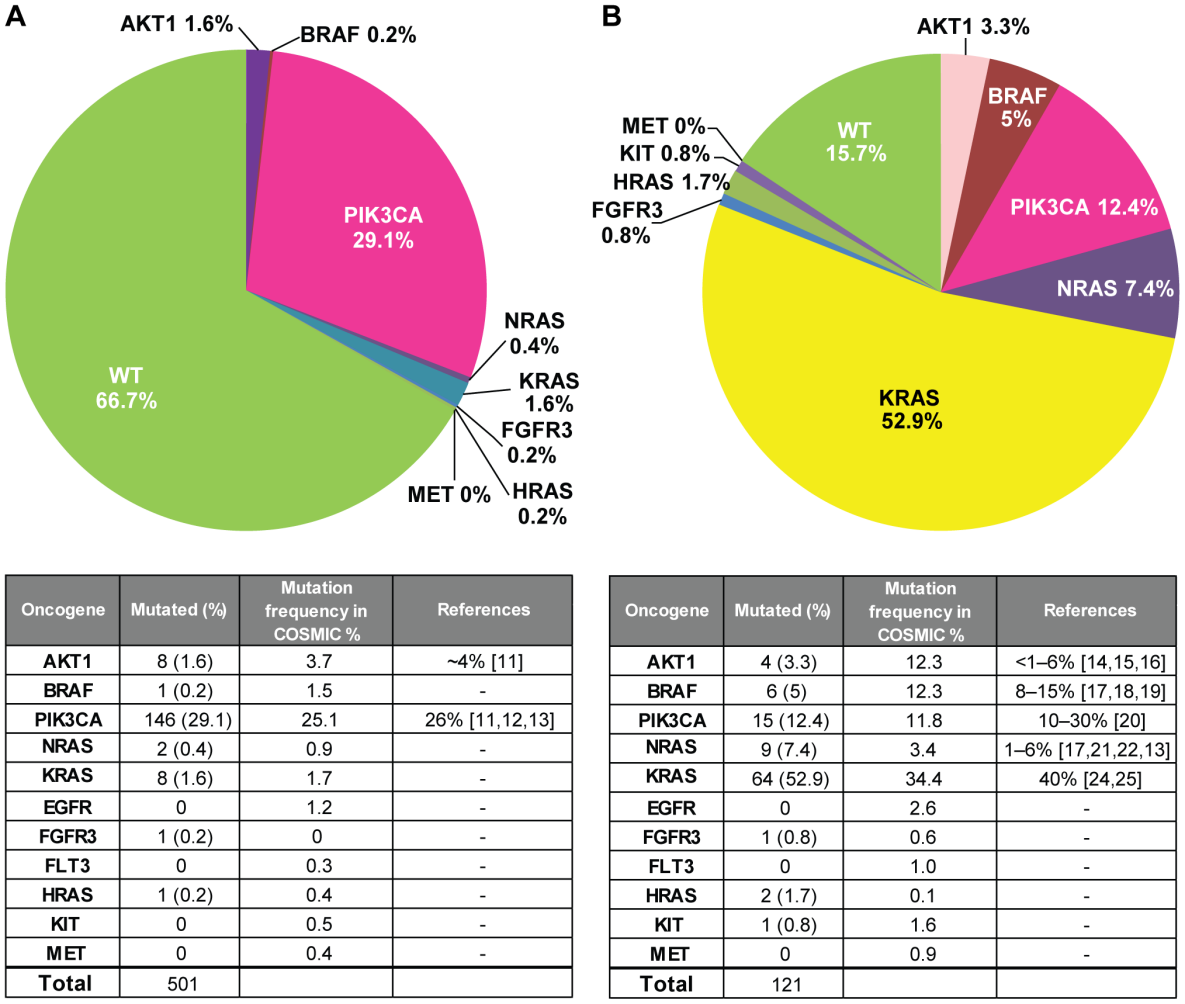
Prevalence of oncogenic mutations detected by MUT-MAP in (A) breast and (B) colorectal tumors compared to COSMIC database and literature citations.

We have performed oncogene mutation profiling on over 1000 individual tumor samples, including FFPE samples, from various cancer types. As an example, using the data generated with MUT-MAP we were able to determine the prevalence of specific mutations in breast and colon cancer (Figure 5A and B, respectively). For a collection of over 500 breast cancer samples we found 29.1% *PIK3CA* mutations, which is consistent with the COSMIC database [15], [16], [17], [18]. We observed many *KRAS* (52.9%), *PIK3CA* (12.4%), and *NRAS* (7.4%) mutations in a colon cancer tissue collection (N = 121). The prevalence of these mutations also correlate well with those listed in the COSMIC database and other literature [19], [20], [21], [22], [23], [24], [25], . These results show that MUT-MAP is a sensitive and accurate platform to determine the mutational status in FFPE tissues and may be utilized to classify patients in clinical trials who may derive greater benefit with a targeted therapy.

## Discussion

Targeted therapies based on the mutational profiles of the tumor have become increasingly important in cancer diagnostics. We report here an updated MUT-MAP with expanded mutational coverage that includes 120 hotspot mutations in eleven cancer related genes. This panel requires as little as 2 ng of high quality gDNA from fresh frozen tissues or 100 ng of gDNA from FFPE tissues and validation using mutant plasmids showed robust assay signal and low cross-reactivity with all of the newly added assays. Mutation calls in cell lines were found to be consistent with the COSMIC database and MUT-MAP showed a 0.45% sensitivity in FFPE samples.

In comparison to the SuraSeq500 panel we have demonstrated that MUT-MAP is more sensitive in detecting the *HRAS* Q61R mutation in FFPE samples and has a similar sensitivity for detecting *AKT1* E17K, *KRAS* G12A, D, and G13D mutations. SuraSeq500 was more sensitive in detecting *BRAF* V600E and *EGFR* L858R. Furthermore, MUT-MAP was able to detect these mutations with a much shorter turnaround time from start to finish, including data analysis, than the NGS platform used. While MUT-MAP lacks the breadth of coverage and flexibility of NGS, the platform can accurately and reliably detect hotspot mutations down to 0.45% (*KRAS* G12A) with very little FFPE DNA input. To date, we have utilized the platform to support multiple clinical programs and to study the prevalence of mutations in various disease settings to assist decision-making in drug development.

In conclusion, we describe here the development and validation of MUT-MAP, a high-sensitivity microfluidics chip-based mutation analysis panel to assay 120 hotspots across eleven oncogenes. This panel can rapidly and accurately determine the mutation status of cancer patient samples in a cost-effective and high-throughput manner. The mutation profiling data generated by MUT-MAP can be used to guide clinical decision-making and inform future clinical trial designs that could aid in the development of personalized health care.

## Supporting Information

Figure S1 **Evaluation of assay sensitivity and linearity.**
(TIF)

Table S1 **The preamplification primer sequences for the new MUT-MAP content: oncogenes** ***PIK3CA*** **,** ***HRAS*** **,** ***FGFR3*** **,** ***FLT3*** **,** ***KIT*** **and** ***MET*** **.**
(XLSX)

Table S2 **Cross-reactivity matrix for the newly added assays in** ***FGFR3*** **,** ***FLT3*** **,** ***KIT*** **, and** ***MET*** **.**
(XLSX)
