# Correction: Next Generation MUT-MAP, a High-Sensitivity High-Throughput Microfluidics Chip-Based Mutation Analysis Panel

**DOI:** 10.1371/journal.pone.0096019

**Published:** 2014-04-24

**Authors:** The PLOS ONE Staff

**Notice of Republication**

This article was republished on April 2, 2014, to replace incorrectly published tables in the online version of the article. The publisher apologizes for the errors. Please download Tables 1, 2, and 3 again to view the correct versions.
