# Supplementary figures and images for: Next Generation MUT-MAP, a High-Sensitivity High-Throughput Microfluidics Chip-Based Mutation Analysis Panel
Source: PLoS One. 2014 Mar 21;9(3):e90761. doi: 10.1371/journal.pone.0090761 (PMC3962342; doi:10.1371/journal.pone.0090761)

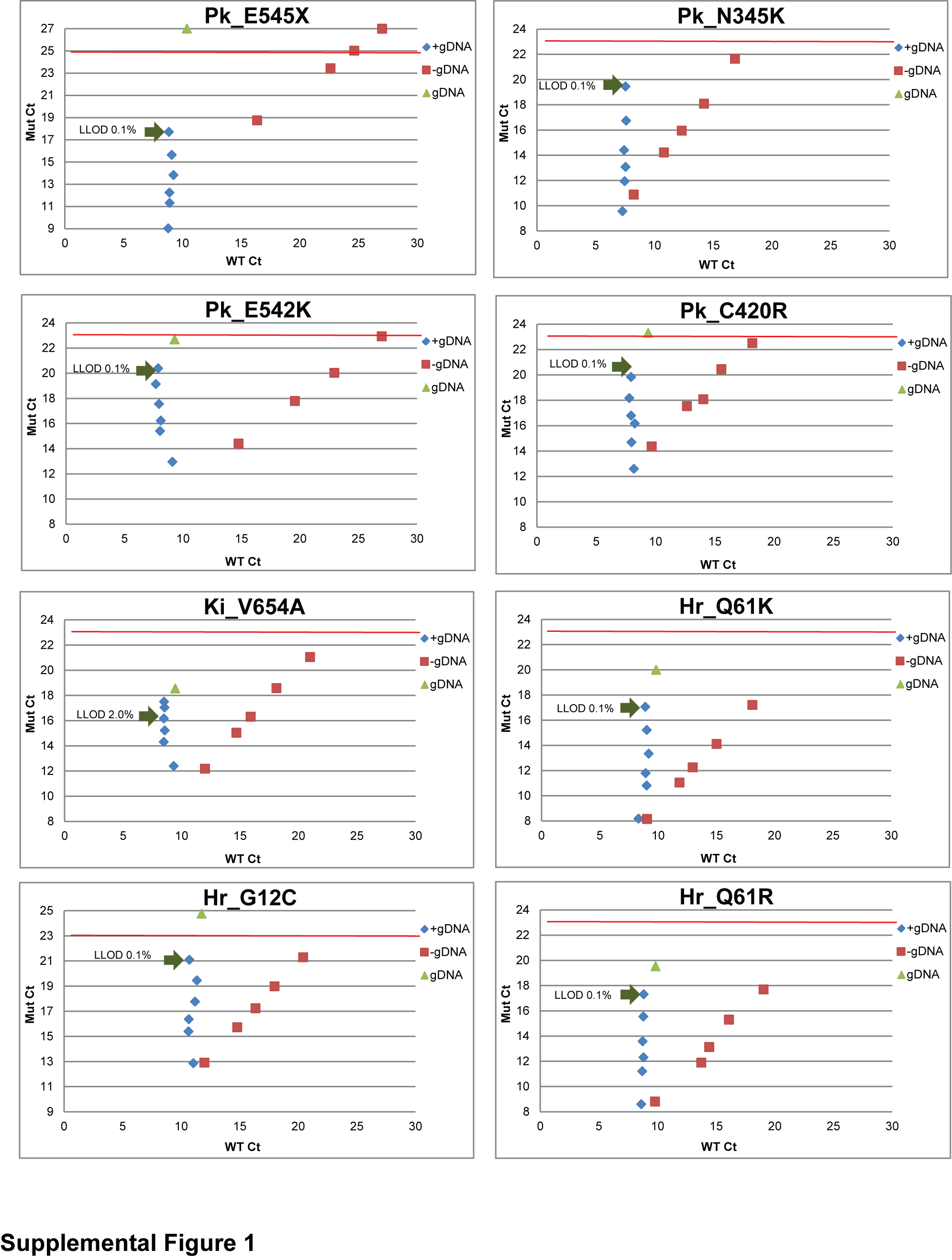

Supplement: Figure S1 — Evaluation of assay sensitivity and linearity. (TIF) [file pone.0090761.s001.tif]
